# *In vivo *reorganization of the actin cytoskeleton in leaves of *Nicotiana tabacum *L. transformed with plastin-GFP. Correlation with light-activated chloroplast responses

**DOI:** 10.1186/1471-2229-9-64

**Published:** 2009-05-29

**Authors:** Anna Anielska-Mazur, Tytus Bernaś, Halina Gabryś

**Affiliations:** 1 Department of Plant Physiology and Biochemistry, Faculty of Biochemistry, Biophysics and Biotechnology, Jagiellonian University, Gronostajowa 7, 30-387 Kraków, Poland; 2 Department of Plant Anatomy and Cytology, Faculty of Biology and Environmental Protection, Silesian University, Jagiellońska 26/28, 40-032 Katowice, Poland

## Abstract

**Background:**

The actin cytoskeleton is involved in the responses of plants to environmental signals. Actin bundles play the role of tracks in chloroplast movements activated by light. Chloroplasts redistribute in response to blue light in the mesophyll cells of *Nicotiana tabacum*. The aim of this work was to study the relationship between chloroplast responses and the organization of actin cytoskeleton in living tobacco cells. Chloroplast movements were measured photometrically as changes in light transmission through the leaves. The actin cytoskeleton, labeled with plastin-GFP, was visualised by confocal microscopy.

**Results:**

The actin cytoskeleton was affected by strong blue and red light. No blue light specific actin reorganization was detected. EGTA and trifluoperazine strongly inhibited chloroplast responses and disrupted the integrity of the cytoskeleton. This disruption was reversible by Ca^2+ ^or Mg^2+^. Additionally, the effect of trifluoperazine was reversible by light. Wortmannin, an inhibitor of phosphoinositide kinases, potently inhibited chloroplast responses but did not influence the actin cytoskeleton at the same concentration. Also this inhibition was reversed by Ca^2+ ^and Mg^2+^. Magnesium ions were equally or more effective than Ca^2+ ^in restoring chloroplast motility after treatment with EGTA, trifluoperazine or wortmannin.

**Conclusion:**

The architecture of the actin cytoskeleton in the mesophyll of tobacco is significantly modulated by strong light. This modulation does not affect the direction of chloroplast redistribution in the cell. Calcium ions have multiple functions in the mechanism of the movements. Our results suggest also that Mg^2+ ^is a regulatory molecule cooperating with Ca^2+ ^in the signaling pathway of blue light-induced tobacco chloroplast movements.

## Background

Actin cytoskeleton (AC) provides tracks for myosin-mediated movements of organelles in plant cells [1]. The dynamic nature of the cytoskeleton depends on actin-binding proteins which control the assembly of actin filaments (AFs) and their organization into higher-order structures [2].

On the basis of AC, chloroplasts change their intracellular arrangement in response to light. These movements are controlled only by blue light in higher plants [3]. Weak blue light (wBL) induces an accumulation response in which chloroplasts gather along the cell walls perpendicular to the light direction. Strong blue light (SBL) induces an avoidance response in which they stay at the walls parallel to the light direction, away from the most illuminated parts of the cell. The light signal is perceived by phototropins (phot1 and phot2), blue-light photoreceptors localised at the plasma membrane [4,5]. Both phototropins mediate chloroplast accumulation, whereas phot2 mediates the avoidance response [6,7]. Chloroplasts of several algae, mosses, ferns and aquatic angiosperms respond also to red light [8,9].

Chloroplasts move along AFs using myosins associated with their membrane [10-12]. Microtubules do not seem to be involved in the directional redistribution of chloroplasts in higher land plants [1,13]. In spite of recent advances little is known about the pathway upon which the blue light signal is transmitted from phototropins to the motor apparatus (see reviews [14,15]). Only two types of secondary messengers have been critically discussed in this context: Ca^2+ ^ions and the phosphoinositide kinases.

Calcium ions regulate the activity of many cytoskeletal proteins and act as secondary messenger in several plant signalling pathways including those initiated by phototropins [16-19]. As shown in studies employing the aequorin Ca^2+ ^reporter system, BL acting through phot1 induced an increase in cytosolic Ca^2+ ^in *Arabidopsis *and tobacco seedlings [20]. Phototropin 1 was also responsible for triggering an influx of Ca^2+ ^across the plasma membrane in *Arabidopsis *seedling hypocotyls [21] and for activating Ca^2+ ^channels at the plasma membrane of *Arabidopsis *mesophyll protoplasts [22]. Calcium ions have been postulated as a potential secondary messenger in red light-controlled chloroplast movements and cytoplasmic streaming in the aquatic angiosperm, *Vallisneria gigantea *[23]. The function of Ca^2+ ^in BL-induced movements still awaits clarification. Manipulating cytosolic calcium homeostasis with various calcium antagonists was shown to interfere with both wBL and SBL chloroplast responses [24,25]. However, this does not explain the role calcium ions play in their mechanisms.

A second category of potential secondary messengers has been proposed, based on different effects of wortmannin, an inhibitor of phosphoinositide-3-kinases, on accumulation and avoidance chloroplast responses in the duckweed *Lemna trisulca *[26]. The authors put forward a model linking the phosphoinositide kinases and other phosphoinositide cycle enzymes with light signal transduction. According to this model the direction of chloroplast movements is determined by phosphoinositides, whereas Ca^2+ ^ions are required only to control the activity of the motor apparatus.

In the last few years we have sought a target of the phototropin-mediated signal which initiates the chloroplast redistribution. In the red-sensitive species actin cytoskeleton was shown to play that role for the phytochrome-mediated signaling [8,9]. Our recent results point to myosin rather than to actin as the target in blue-sensitive higher plants [12,27]. Up till now, the light effects on AC were studied using fixed tissue. Here, we attempted to visualize the cytoskeleton in living mesophyll cells. The truncated plastin-GFP construct [28] was successfully expressed in mature tobacco leaves giving a stable, fully functional transgenic line. The objective of the present study was to perform life imaging of the actin dynamics in this transgenic *Nicotiana tabacum *system, and to take a step toward identifying secondary messengers and relations between them in blue light-controlled chloroplast movements. To achieve the latter goal we compared the effects of calcium agonists/antagonists and of wortmannin on AC and on the chloroplast responses.

## Results

### Characteristics of the transgenic tobacco line

**Figure 1:**
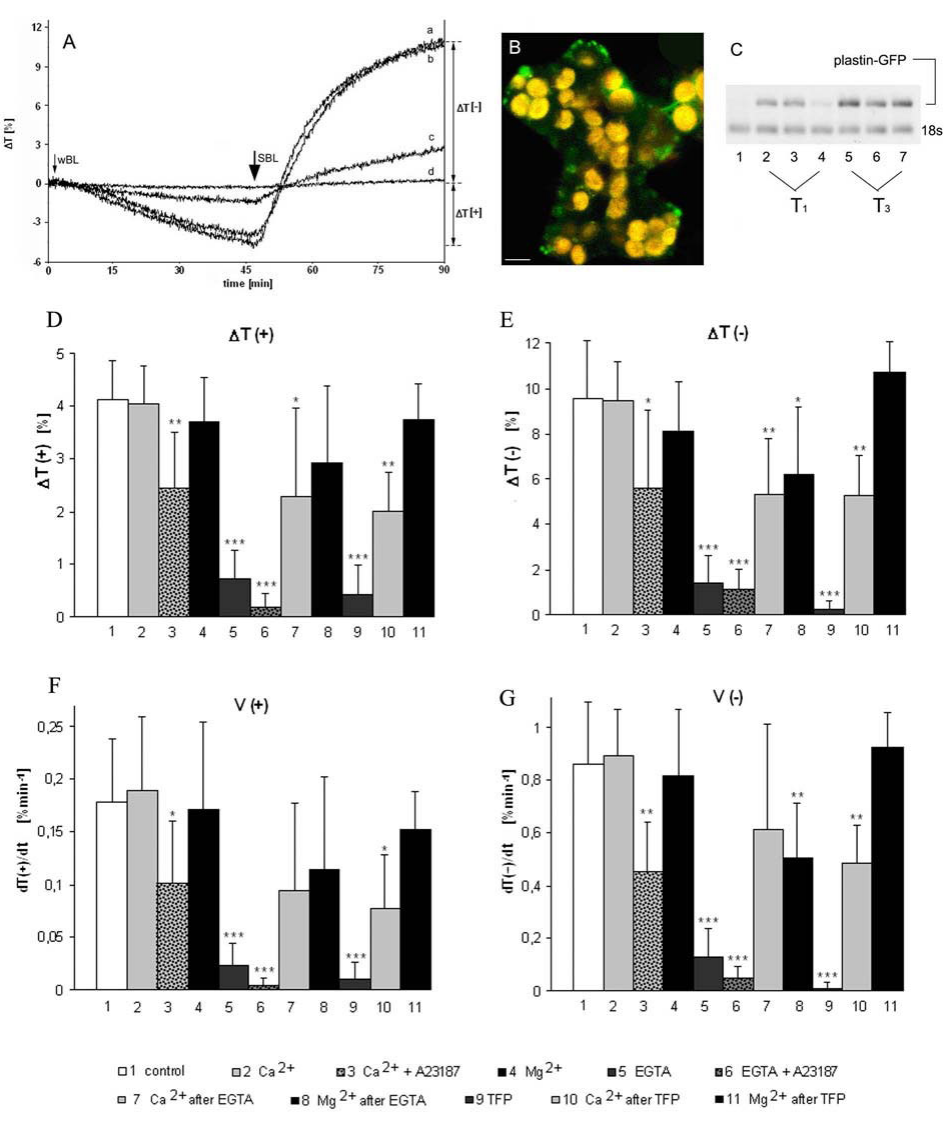
**(A) Chloroplast responses to blue light in wild-type and transgenic *N. tabacum *cv. Samsun expressing plastin-GFP**. The curves show changes in transmission of red measuring light (ΔT) through dark-adapted leaves exposed to continuous weak blue light (wBL, 0.4 Wm^-2^, 45 min) and strong blue light (SBL, 10 Wm^-2^, 45 min). ΔT [+] and ΔT [-] denote amplitudes of accumulation and avoidance responses respectively. Representative responses for about seventy tests carried out with three-month-old wild-type (a) and transgenic (b) tobacco plants. Curves c and d are representative of two-month (c) and one-month-old (d) plants. **(B) Actin organization in immature mesophyll of transgenic (one-month-old) plants grown *in vitro***. Bar, 10 μm. **(C) RT-PCR**. (1) control, non-transformed plant, (2–4) three plants of T_1 _generation, (5–7) three plants of T_3 _generation. **(D – G) Parameters of blue light-controlled chloroplast responses in mature leaves of transgenic *N. tabacum***. (D, E) Amplitudes: ΔT(+) of weak (wBL, 0.4 Wm^-2^), and ΔT(-) of strong (SBL, 10 Wm^-2^) blue light responses. (F, G) Velocities: V(+) of wBL, and V(-) of SBL responses. Averages of 7–14 measurements. Error bars represent SD. Asterisks denote the significance of differences (p-value calculated with the unpaired t-test, * p = 0,05–0,001; ** p = 0,001–0,0001; *** p < 0,0001).

The expression of plastin-GFP did not affect the responses of chloroplasts in *Nicotiana tabacum*. The amplitudes and kinetics of these responses were about the same in both transformed and non-transformed three-month-old plants (Fig. 1A: a, b). Notably, the chloroplast redistribution was much weaker in younger plants grown *in vitro *for up to two months after each passage (Fig. 1A: c, d). Instead of filamentous structures present in three-month-old plants, fluorescent speckles and diffuse fluorescence were observed throughout the young tissue (Fig. 1B). Obviously, the actin tracks necessary for chloroplast movements were undeveloped in young plants.

The expression of plastin-GFP was generally low, with varying levels in different cells and tissues. Two plant generations were screened for the most uniform expression of plastin-GFP in the spongy mesophyll cells. The best plant was reproduced vegetatively and used in further investigations. The varied expression of plastin-GFP between plants coming from different generations, observed in the confocal images at the protein level, was confirmed by RT-PCR at the level of mRNA (Fig. 1C). No differences in germination, development and flowering were found between transgenic and wild type plants. Also the efficiency of photosynthesis measured as *in vivo *chlorophyll fluorescence was identical in the two groups (results not shown).

### General outline of experiments

Samples from one leaf, subjected to identical treatment, were concomitantly used for measuring the movement activity of chloroplasts and for testing AC by confocal microscopy. Chloroplast movements were activated with blue light. To seek BL-specific effects on the organization of AC, the samples tested microscopically were irradiated with blue or red light. Red light, inactive in chloroplast redistribution, was used as control. The effects of compounds disturbing calcium homeostasis (EGTA alone or supported by calcium ionophore, trifluoperazine) were compared in the above two experimental settings, and then Ca^2+ ^ions were added to counteract the antagonists. Because earlier investigations showed that Mg^2+ ^could eliminate the inhibitory effects of EGTA on chloroplast redistribution [23], all experiments were also repeated with Ca^2+ ^substituted by Mg^2+^. Visualization of changes in the cytoskeleton was supported by quantitative image analysis. Additionally, a potential interaction between Ca^2+ ^and phosphoinositide-mediated signal transduction pathways was tested by compensating wortmannin inhibitory effects on chloroplast responses with addition of both divalent cations.

### Actin organization in continuous light

**Figure 2:**
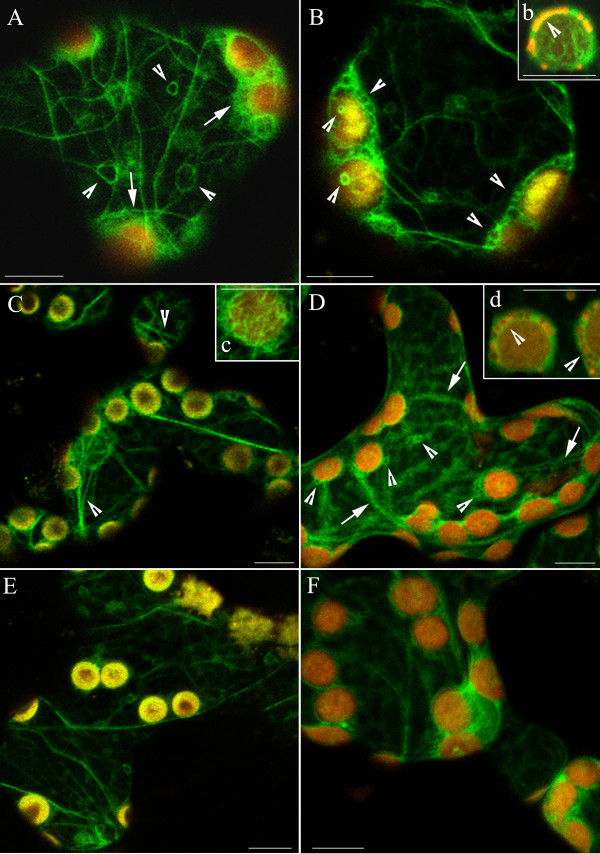
**The actin cytoskeleton in 3-month-old tobacco mesophyll cells as visualized by plastin-GFP (green fluorescence)**. (A, B) Network of actin in dark-adapted cells. "Baskets" around chloroplasts marked with arrows and circular structures marked with arrowheads; yellow-red colour comes from autofluorescence of chloroplasts. (C) Reorganization of F-actin after 1 h of exposure to continuous wBL (0.4 Wm^-2^). Strands which spread across the cortical cytoplasm, frequently split into thinner filaments (marked with arrowheads). (c) Actin filaments forming baskets are better resolved after wBL. (D) Wide bands of F-actin (arrows) have a loose contact with chloroplasts after exposure to SBL (10 Wm^-2^, 20 min). (E) Effect of continuous wRL (0.24 Wm^-2^, 1 h) or (F) to SRL (6.7 Wm^-2^, 20 min) on the actin cytoskeleton. Scale bars, 10 μm. The cytoskeleton forms numerous small loops (B and D, arrowheads), most of them containing mitochondria. Insets b and d show magnified chloroplasts with mitochondria visible (orange/red colour) in the AC loops after staining with TMRE.

In dark-adapted cells distinctly outlined actin bundles formed a branched network (Fig. 2A, B). The chloroplasts were associated with basket-shaped structures consisting of thin AFs (arrows). These baskets were tightly bound together around adjacent chloroplasts and attached to cortical actin bundles. Circular structures of various sizes were sporadically seen in the cytoplasm (Fig. 2A, arrowheads). Numerous small loops were present, predominantly on the surface of chloroplasts (Fig. 2B, arrowheads). Most of them contained mitochondria (Fig. 2b arrowhead).

**Figure 3:**
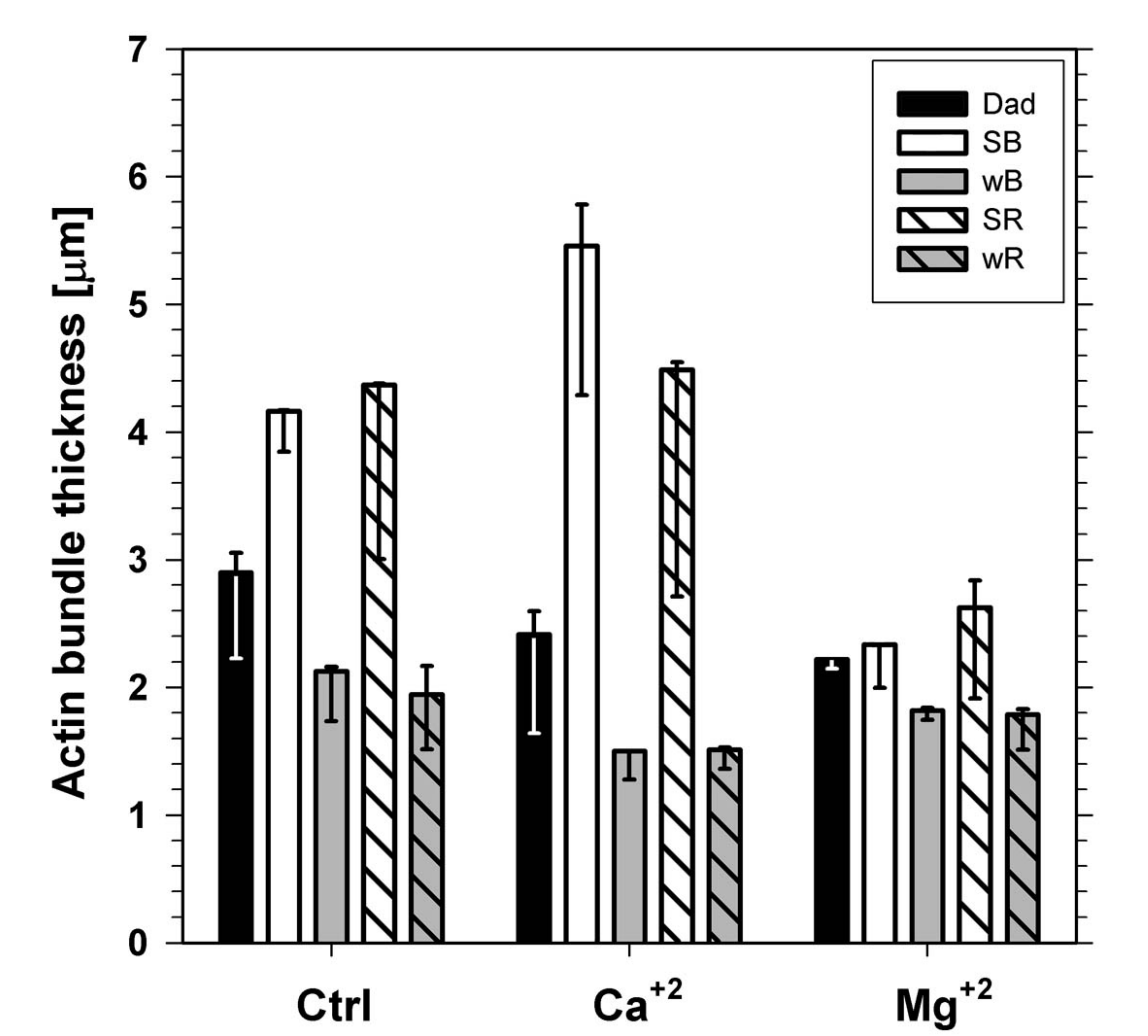
**Thickness of actin bundles in control (Ctrl) and in the presence of calcium (Ca^+2^) and magnesium (Mg^+2^) ions**. The bundle thickness was measured in cells adapted to darkness (Dad, black bars) and in cells illuminated with strong (SB) or weak (wB) blue light (empty and gray bars, respectively) and with strong (SR) or weak (wR) red light (backslashed and backslashed gray bars, respectively). The thickness was calculated in micrometers (μm) as 99th percentile of the data corresponding to averages of optical sections, whereas error bars represent 95% confidence intervals.

The organization of AC was modified after irradiation with wBL. This light induced an accumulation response of chloroplasts, shown in Fig. 1A and 1D as decrease of light transmission ΔT(+) through the leaf. The AFs reorganized without losing their clear-cut appearance (Fig. 2C). The quantitative analysis showed a distinct narrowing of actin bundles in weak light (Fig. 3). Single chloroplasts were wrapped in discrete bundles finer than those present in the dark (Fig. 2c; for quantitative evaluation see Additional file 1, Ctrl: Energy of actin distribution pattern in F-actin baskets surrounding chloroplasts). The general distribution of mitochondria with respect to chloroplasts did not change as compared with the dark-adapted material (not shown).

After the tissue was exposed to SBL the image of almost all cell actin became diffuse with occasional single wide strands to which chloroplasts were attached (Fig. 2D, arrows). The chloroplasts took on the profile position characteristic of the avoidance response (ΔT [-] in Fig. 1A and 1E, Additional file 2). The baskets on chloroplast surfaces became diffuse but the small loops containing mitochondria were still conspicuous (Fig. 2d). The connection between the baskets and the actin network appeared looser than in the dark-adapted or wBL-treated cells. The F-actin image started to get diffuse as early as several min after SBL irradiation even if the tissue had been pre-irradiated with wBL. The wide strands were seen reorganizing upon irradiation with strong light (Additional files 3, 4 and 5). The "diffusion effect" was reversible and the reconstruction of a distinct, branched actin network took place in the SBL-irradiated samples subsequently treated with continuous wBL (Additional file 6). This reconstruction was observed no sooner than 60 min after the onset of weak light exposure.

The effects of blue light were compared with those produced by red light (RL). Almost the same images of the actin network were obtained after exposure to BL or RL with equivalent quantum fluxes (Fig. 2E, F). Strong light produced indistinguishable images of widened F-actin bundles and foamy chloroplast baskets irrespective of wavelength. Hence, no blue-specific differences could be detected in the actin structure. The quantitative results in Fig. 3 (Ctrl) and Additional file 1 (Ctrl) support this observation.

### Effects of Ca^2+^, Ca^2+ ^+ ionophore A23187 and Mg^2+^

**Figure 4:**
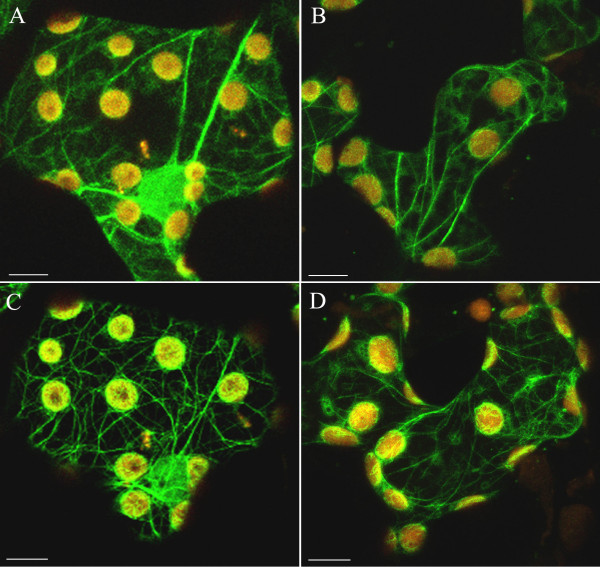
**Influence of calcium and magnesium ions on the actin network in dark-adapted and weak light-irradiated cells**. Samples were incubated for 2 h with 5 mM Ca^2+ ^(A, C) or 5 mM Mg^2+ ^(B, D). Actin cytoskeleton in the dark-adapted cells (A, B) and after irradiation with continuous weak red light for 1 h (C, D). Scale bars, 10 μm.

The effect of extracellular calcium on the structure of the actin cytoskeleton and on chloroplast movements was studied using a 5 mM solution of calcium nitrate. The image of AC became more distinct after the addition of Ca^2+^. The effect was visible in the dark-adapted mesophyll cells and even stronger after wBL or wRL irradiation (Figs 3, 4A and 4C). Photometric transmission changes reflecting the chloroplast responses to BL were about the same as with the control (Fig. 1D–G, bar 2). Exposure of the of Ca^2+^-treated tissue to SBL and SRL produced similar wide bands of AFs with side adhering chloroplasts. All formations observed in these images looked very similar to those from SL-irradiated controls (Fig. 3, Additional file 7).

The concomitant application of Ca^2+ ^ionophore A23187 worsened the cytoskeleton image typical of the ambient Ca^2+^-solution, particularly after exposure to either wBL or wRL (data not shown). The addition of ionophore A23187 to the calcium nitrate solution reduced amplitudes by about 40% and velocities by about 50% in both chloroplast responses (Fig. 1D, G, bar 3).

As shown in Fig. 4 the actin cytoskeleton at higher extracellular Mg^2+ ^concentration looked similar to that at the presence of Ca^2+ ^both in dark-adapted and wL-irradiated tissue. The tendency to widening was occasionally visible in strong light (see Additional file 6) but the quantitative analysis showed that Mg^2+ ^ions subdue the strong light effect (compare striped bars in Fig. 3). The parameters of chloroplast responses were somewhat lower with Mg^2+ ^than with either Ca^2+ ^or the control, however, the difference was statistically insignificant (Fig. 1D–G, compare bars 4 and 2).

### Effects of EGTA and EGTA + calcium ionophore A23187

**Figure 5:**
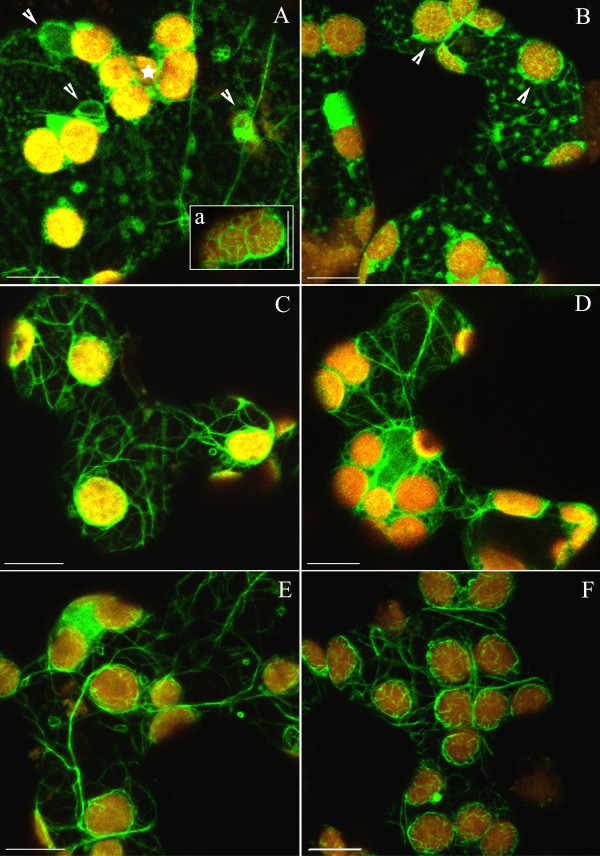
**Disintegration of F-actin in EGTA and restoration of actin network prompted by calcium or magnesium ions**. (A) Formation of actin foci in response to 0,5–1 h incubation with 1 mM EGTA in dark-adapted cells. Fluorescent spots and loops of various sizes (arrowheads) are visible throughout the cytoplasm. Chloroplasts are arranged into tight clusters (asterisk). Thin filaments are present on chloroplast surfaces (a). (B) Actin foci persist after wBL irradiation. Distinct baskets around chloroplasts (arrowheads) became more visible after exposure to weak light. Effect of 5 mM Ca^2+ ^(C, D) or 5 mM Mg^2+ ^(E, F), each applied for 2 h on actin organization in EGTA pre-treated cells. In both cases, F-actin network recovered in dark-adapted cells (C, E) and after additional exposure to continuous wBL for 1 h (D, F). Scale bars, 10 μm.

Potent inhibition of chloroplast responses concomitant with dramatic changes in AC organization were observed in tissue incubated with 1 mM EGTA for 30 min to 1 h in the dark (Fig. 1D–G, bar 5; Fig. 5A). EGTA caused the formation of a characteristic spotted pattern all over the cell. At the same time, a dense network of fine AFs formed at the chloroplast surfaces (Fig. 5a). Loops of various sizes with a fluorescent-green tint inside were visible in some cells (Fig. 5A, arrowheads). Numerous chloroplasts were grouped into tight clusters (asterisk). After exposure to weak light, the spotted pattern persisted but the chloroplast baskets became clearly visible: very fine filaments appeared on the surfaces of chloroplasts (Fig. 5B, arrowheads). The energies of F-actin patterns corresponding to chloroplast baskets were 33.7 for dark-adapted vs 37.1 for wB (99th percentile, arbitrary units). The increase in energy signifies growing inhomogeneity of AC i.e. the emergence of distinct AFs. This difference was detectable at all percentiles (not shown). The structure characteristic of treatment with EGTA disappeared in strong light. Simultaneously, filamentous actin reappeared in the cells in the form of a few widened strands (Additional file 7C and 7D).

When EGTA was applied together with the calcium ionophore, both accumulation and avoidance responses were almost eradicated (Fig. 1D–G, bar 6). Strong inhibition of the chloroplast movements was observed as early as 30 min after the beginning of incubation. Along with the arrest of movement, the addition of the calcium ionophore accelerated and intensified changes in the AC organization produced by EGTA (cf. Fig. 5 and Additional file 8).

### Reversion of EGTA effects by Ca^2+ ^and Mg^2+^

Calcium and magnesium ions reversed the damage caused by EGTA in the dark-adapted cells when used directly after the chelating agent (Fig. 5C, E). Irradiation with wL helped the reconstruction of the filamentous structure of actin. Sharper AFs were restored in the presence of Mg^2+ ^than in the presence of Ca^2+ ^(Fig. 5D, F). Both ions caused the chloroplasts to separate from the EGTA-induced clusters (Fig. 5C–F). While the structure of the actin network was considerably improved by Ca^2+^, the chloroplast responses were reactivated only by 50% (Fig. 1D–G, bar 7). As with its effect on the cytoskeleton, Mg^2+ ^restored both chloroplast responses to blue light somewhat better than Ca^2+ ^(Fig. 1D–G, bar 8, contrast with 7).

### Effects of trifluoperazine and their reversion by Ca^2+ ^and Mg^2+^

**Figure 6:**
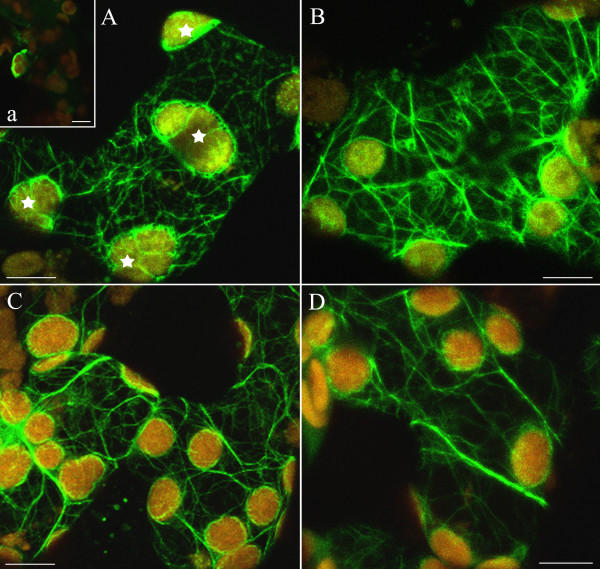
**Disintegration of actin bundles by trifluoperazine and its reversal by Ca^2+ ^and Mg^2+^**.(A) Images of disordered F-actin after treatment with 20 μM TFP for 30 min; chloroplast clusters marked with asterisks. Inset (a): effect of 1 h treatment with TFP in darkness. (B) Recovery of actin bundles by continuous wBL (1 h). The irradiation started 15 min after the onset of TFP treatment. The complete AC reconstruction in dark-adapted mesophyll cells pre-treated with TFP for 30 min and thereafter incubated with 5 mM Ca^2+ ^or 5 mM Mg^2+ ^for 2 h (C, D, respectively). Scale bars, 10 μm.

Trifluoperazine (TFP, 20 μM), a blocker of calmodulin caused destabilization of the AC as early as 15 min after application. Chloroplast clusters formed in most cells as with EGTA (Fig. 6A, compare with Fig. 5A, asterisks). AC-associated fluorescence disappeared after 1 h of incubation in the dark (Fig. 6a). Early exposure to light, especially wBL, brought about a reconstruction of actin bundles and chloroplast separation (Fig. 6B). In spite of AC recovery, the chloroplasts did not respond to light in the TFP-treated tissue. The first disturbances were detected after 15 min, and 15 min later the avoidance response was practically extinguished. Both responses were completely inhibited after 45 min treatment with TFP (Fig. 1D-G, bar 9).

The effect of TFP was reversed by calcium and magnesium in the dark (Fig. 6C, D). Both ions caused the chloroplasts to separate from the TFP-produced clusters. Even though both ions prompted the recovery of chloroplast movements, magnesium was notably twice as effective as calcium in the reactivation (Fig. 1D-G, bar 11, contrast with 10).

Irrespective of the ionic/pharmacological treatment, the effects of BL and RL on cortical AC were comparable. Irradiations with equivalent quantum fluxes of SB and SR and/or wB and wR resulted in formation of F-actin patterns of similar energies, respectively. No blue-specific differences could be detected (see Additional file 1).

### Effects of wortmannin

**Figure 7:**
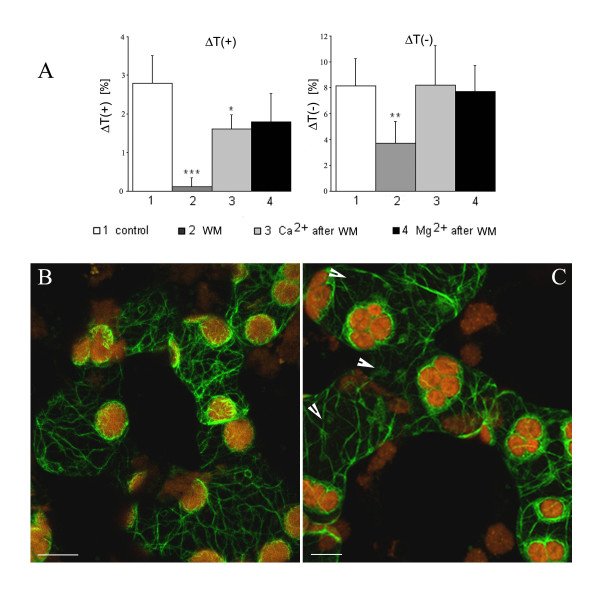
**Effect of wortmannin (WM) on BL-induced chloroplast responses and on the actin cytoskeleton in tobacco leaves**. (A) Amplitudes: ΔT(+) of weak (wBL, 0.4 Wm^-2^), and ΔT(-) of strong (SBL, 10 Wm^-2^) blue light-induced responses after 1.5 h incubation with 10 μM WM. The leaves were subsequently treated with 5 mM Ca^2+ ^or Mg^2+ ^for 3 h (right columns). Averages of 5–7 measurements. Error bars represent SD. Asterisks denote the significance of differences (p-value calculated with the unpaired t-test, * p = 0,05–0,001; ** p = 0,001–0,0001; *** p < 0,0001). The network of actin bundles in the mesophyll cells of the transgenic tobacco after 1,5 h incubation with 10 μM (B), and 50 μM (C) WM. Note the chloroplast clustering and disturbances of AC integrity (arrowheads) at the higher WM concentration. Scale bars, 10 μm.

Wortmannin (WM), an inhibitor of phosphoinositide-3-kinase, had a dramatic effect on chloroplast movements at a concentration of 10 μM (Fig. 7A). The accumulation response was eliminated and the avoidance response was reduced by half after 1.5 h exposure. Again, Ca^2+ ^and Mg^2+ ^negated the inhibition, with full recovery of the avoidance response obtained with both investigated ions. The influence of WM was stronger on velocities than on amplitudes, but they were similarly reactivated by Ca^2+ ^and Mg^2+ ^(data not shown). In this case, however, the inhibitory effect on the movement was not reflected in the shape of cellular actin: AC remained completely unaffected by WM (Fig. 7B). Only when the concentration was increased to 50 μM did some perturbations in the continuity of actin bundles become perceptible (Fig. 7C). This higher concentration of WM abolished both chloroplast responses to light.

## Discussion

The widening of actin strands upon SL-irradiation can be interpreted as a relaxation of the structure of actin bundles. It might be a result of some yet undefined interaction between filaments (or between actin and ABPs) in SL, leading to the formation of looser bundles. The differences between the organization of cytoskeleton in wL and SL may have consequences for the manner in which actin anchors the chloroplasts in a cell under different light conditions. The tendency of chloroplasts to be displaced during centrifugation was investigated in *Lemna trisulca*, a model species used in studies on blue-activated chloroplast movements in higher plants. The ability of chloroplasts to resist centrifugal force depended on light pre-treatment. Whereas wL anchored chloroplasts in the cells, SL loosened the binding and made them easier displaceable by centrifugal forces [29]. The relaxation reported currently might be the basis of the mentioned SL effect.

Circular forms of AFs sometimes occurred in dark-adapted or wBL-treated tobacco cells. Similar forms had previously been observed in fixed *Adiantum *protonemal cells and assigned a role in chloroplast anchoring [30]. However, in *Adiantum *the circles had a much bigger diameter and were found only in irradiated tissue.

The differences observed in tobacco are in contrast to the results obtained with fixed tissue of *A*. *thaliana*, where no structural dissimilarities of actin were detected between leaves treated with wBL or SBL [27]. The different susceptibility of actin to light in *Arabidopsis *as compared to *Nicotiana *might be attributed to a non-identical organization of the filament bundles in these species. This could account for three unsuccessful attempts to transform *Arabidopsis *leaves with the plastin-GFP construct used in this study whereas siliques and sepals showed an effective transformation (unpublished data from our laboratory). On the other hand, the discrepancy between the actin images obtained for SBL-irradiated tobacco and *Arabidopsis *could be a consequence of the fixation procedure used for the latter species.

Seeking BL-specific actin reorganization we compared the effects of blue and red light on the actin cytoskeleton. As with fixed cells, RL and BL effects were similar, even though RL induces no directional chloroplast movement in tobacco [31]. This lack of difference confirms our previous conclusion that the directionality of chloroplast responses is not based on BL-specific changes of F-actin, and that other factor(s) must determine the direction of chloroplast movement.

The presence of the dense cortical F-actin network associated with chloroplast baskets was demonstrated in living tobacco cells as in other reports dealing with fixed actin in *Arabidopsis *[13,27]. The structure of the actin baskets and their interactions with the cortical AC seem to be of key importance for chloroplast positioning in higher land plants. Even though irradiation with wL and/or SL changed the structure of actin baskets in living cells, they were always tightly associated with chloroplast surfaces.

The significant improvement of the cytoskeleton image by Ca^2+ ^and Mg^2+ ^may be due to the binding of these ions to either AFs or plastin. Contrary to our results, external calcium and magnesium had no visible effect on the phalloidin-labelled AC organization in tobacco BY-2 protoplasts [32]. In poppy pollen tubes, F-actin was fragmented into punctate foci at increasing concentration of cytosolic free Ca^2+ ^[33].

The damage to AC made by external EGTA depleting the cytosolic Ca^2+ ^confirms the obvious fact that calcium is important for the maintenance of microfilament integrity. The formation of the well structured filamentous actin on the surfaces of chloroplasts (Fig. 5B) may be due to Ca^2+ ^extraction from these organelles during wBL irradiation. Blue light of 1 μmol m^-2 ^s^-1 ^produced a transient increase in cytosolic Ca^2+ ^in *A.thaliana *leaves, which originated partly from internal calcium stores [5]. Our results can be interpreted in terms of BL causing an efflux of calcium from chloroplasts, and its continuous chelating by extracellular EGTA. This combined action of light and EGTA might have induced the transient formation of fine actin baskets on chloroplast surfaces, whereas EGTA prevented a stable restoration of actin cytoskeleton in the cells.

Along with the destruction of the actin network, EGTA strongly inhibited chloroplast responses in tobacco, similar to the effects previously reported for ferns and water angiosperms [24,25]. Combined treatment with EGTA and A23187 merely accelerated and intensified the effects of EGTA. The ionophore facilitated the efflux of calcium from the cells.

Trifluoperazine, a blocker of calmodulin completely inhibited the chloroplast movements in tobacco, similar to the inhibition previously reported for *Lemna *[24]. TFP also caused the disappearance of almost all AC-related fluorescence after 1 h dark-incubation. Light effected a partial reconstruction of AFs in the presence of TFP, most probably due to an increase in the cytosolic Ca^2+ ^concentration. Remarkably, chloroplast responses were not restored. Even though Ca^2+ ^presumably released by BL efficiently rebuilt the TFP-destroyed AFs, it was not capable of reactivating chloroplast responses. This may suggest that calmodulin per se is involved in transmitting the directional signal.

Both Ca^2+ ^and Mg^2+ ^effectively restored the EGTA- and TFP-damaged actin cytoskeleton. The differences between the actin bundles restored by these ions might be attributed to differences in de novo actin polymerization. In a study on rabbit skeletal muscle, Mg^2+ ^played a stronger role in the mechanism of actin polymerization than Ca^2+ ^due to faster nucleation of Mg-ATP-actin than Ca-ATP-actin [34]. Indeed, Mg-ATP-actin nucleates three orders of magnitude faster than Ca-ATP-actin [35,36]. Besides, actin containing tightly-bound Mg^2+ ^differs structurally and functionally from actin containing tightly-bound Ca^2+ ^[37,38].

Both ions were shown to restore not only the actin cytoskeleton, but also chloroplast responses in EGTA/TFP treated tobacco cells. Ca^2+ ^has previously been reported to restore the EGTA-inhibited chloroplast photo-orientation in *Adiantum *protonemal cells [39]. Mg^2+ ^has been shown to counteract the inhibitory effect of EGTA in *Lemna *when applied together with the chelator [24]. It was hypothesized that Mg^2+ ^blocked the calcium channels through which Ca^2+ ^was removed from the cell by the external EGTA. Extracellular magnesium has indeed been shown to significantly modifiy the transport of all major ions, H^+^, Ca^2+^, and K^+ ^in bean mesophyll cells [40].

Remarkably, Mg^2+ ^was twice as effective in restoring chloroplast photo-responses than Ca^2+ ^in samples pre-treated with TFP. How was Mg^2+ ^able to fully recover the directional chloroplast movement? Could it act indirectly, bypassing calmodulin or triggering other pathways that substitiute for calmodulin activity? It is difficult to answer these questions because magnesium homeostasis is still poorly understood [41]. The molecular details of Mg^2+ ^transport between cellular compartments in plants are still far from clear [42,43]. Mg^2+ ^ions are stored mainly in vacuoles. A large part of the cytoplasmic magnesium is complexed by ATP. The concentration of free Mg^2+ ^in the cytosol must therefore be strictly regulated, which is a pre-condition for playing a role in signal transduction [42]. In animal systems, magnesium has been postulated as acting as an intracellular messenger [41]. Could it play such a role also in plant cells?

The strong inhibition of chloroplast movement by wortmannin shows that the model assigning phosphoinositide kinases a key role in the transduction of the orienting BL signals in *Lemna *[26] may be valid also for higher land plants. On the other hand, the model needs further refinement. Firstly, WM at a concentration of 10 μM, which is strongly inhibitory for both chloroplast responses, had no effect on tobacco AC. Thus, the BL signals are not directed to actin, which is consistent with our former conclusion [12,27]. Small disturbances in the network were perceptible only at 50 μM, above the range of concentrations commonly used in plants [26,44]. Secondly, the striking recovery of WM-inhibited movements obtained with Ca^2+ ^shows that this ion is not only needed for controlling the motor apparatus (myosin) but also that it transmits the signal downstream of the phosphoinositide kinases. Our results suggest therefore further complications in the model of signal transduction.

All investigated remedial activities of extracellular Ca^2+ ^could be also mimicked by Mg^2+^, in most cases even more efficiently. Thus, our results point to the possibility that Mg^2+ ^is a regulatory molecule cooperating with Ca^2+ ^in the signaling pathway of BL-induced tobacco chloroplast movements. It has to be stressed that extracellular Ca^2+^/Mg^2+ ^reactivated the directional movements even though applied non-directionally. Thus, an asymmetric, polar distribution of some yet undisclosed cellular elements with which this(these) ion(s) interact seems to be required to define the direction of chloroplast movements in higher plants.

## Conclusion

The actin cytoskeleton in the mesophyll of tobacco is sensitive to weak and strong light irrespective of its spectral region (blue or red). Thus, the directionality of chloroplast responses is not based on specific blue light-induced changes of F-actin but on other, yet unidentified factor(s).

The structure of the actin baskets surrounding chloroplasts, and their interactions with the cortical actin cytoskeleton appear to be crucial for chloroplast positioning in higher land plants.

The striking recovery of wortmannin-inhibited movements obtained with Ca^2+ ^shows that these ions play at least two roles in the mechanism of the movements: they control the motor apparatus and transmit the light-generated signal downstream of the phosphoinositide kinases.

Our results show for the first time that Mg^2+ ^is a regulatory molecule cooperating with Ca^2+ ^both in the maintenance of the actin network integrity and in the signaling pathway of chloroplast movements in tobacco.

## Methods

### Plant growth conditions

*Nicotiana tabacum *plants (ecotype Samsun) used for experiments were grown on MS medium supplied with Gamborg vitamins, 3% (w/v) sucrose and solidified with 0.8% (w/v) agar. The axenic cultures were kept in a growth chamber (Sanyo MLR-350, Japan) equipped with fluorescent tubes (Sanyo FL 40SS.W/37 and OSRAM L 36W/77 Fluora, Germany). The fluence rate of the fluorescent light was 60 to100 μmol m^-2 ^s^-1^. The photoperiod was 12/12 h and the temperature was 23°C.

### Constructs, plant transformation and bacterial growth conditions

Tobacco was stably transformed using the *Agrobacterium tumefaciens *strain LBA 4404 containing the binary plasmid pBI 121 (for more details see [27]). The plasmid carried a gene coding for a fusion protein consisting of truncated human plastin and smGFP (plastin-GFP) under the control of the cauliflower mosaic virus 35S promoter. The *Agrobacterium *was cultured for two days in the dark at 28°C in LB, a liquid medium supplemented with 20 mg l^-1 ^rifampicin and 100 mg l^-1 ^streptomycin. Bacteria (OD_600 _1.6) were resuspended in 5 ml of liquid medium containing MS salts supplemented with Nitsch vitamins, 0.2% (w/v) glucose, 0.004% (w/v) adenine, 1.0 mg l^-1 ^BAP and 0.1 mg l^-1 ^NAA. Tobacco leaf discs were inoculated with bacterial suspension and co-cultured for two days in the dark at 28°C in the same medium solidified with agar. The regeneration/selection was carried out on a medium containing MS salts with Gamborg vitamins, 3% (w/v) sucrose, 40 mg l^-1 ^adenine, 1 mg l^-1 ^BAP, 0.1 mg l^-1 ^NAA, carbenicillin to kill the bacteria and kanamycin to inhibit growth of non-transformed plant cells. The carbenicillin content was gradually reduced from 500 to 200 mg l^-1^, while the kanamycin concentration was kept at 50 mg l^-1^. The transformed cells grew into callus and differentiated into shoots via organogenesis. Two generations of transgenic plants were tested for the presence of plastin-GFP under a confocal microscope. One plant exhibiting the most distinct and uniform expression in the mesophyll cells was reproduced vegetatively and cultured under axenic conditions. Three-month-old transgenic leaves of these plants were used for experiments. The vegetative culture was continued for 15 months with plants transferred to fresh medium every 3 months.

Control and transgenic plants obtained from first and third generation seeds were used for RT-PCR. Total RNA obtained with RNeasy Plant Mini Kit (Qiagen GmbH, Germany) and decontaminated from DNA with DNA-freeTM Kit (Ambion Europe Ltd UK) was used for cDNA synthesis with random hexamer primers (RevertAidTM First Strand cDNA Synthesis Kit; Fermentas UAB, Lithuania). The semi-quantitative RT-PCR was carried out after normalization with QuantumRNATM 18S RNA (Ambion Europe Ltd UK, 3:7 primer:competimer ratio), an internal control. Primers were designed using Biology WorkBench 3.2 (plastin-GFP left primer 5'-CTGACATTGAATTAAGCAGGAATG-3' and right primer 5'-AAGCATTGAACACCATAAGTGAAA-3').

### Treating solutions

All solutions were buffered with 10 mM PIPES, piperazine-1,4-bis(2-ethanesulphonic acid). The following treating solutions were used: 5 mM Ca(NO_3_)_2_; 5 mM Mg(NO_3_)_2_; 10 μM calcium ionophore A23187 (4-bromocalcimycin A23187); calcium-free solution: 1 mM EGTA+1.5 mM KH_2_PO_4_+5 mM KNO_3_; 20 μM trifluoroperazine; 10 μM and 50 μM wortmannin. The ionophore and wortmannin were initially dissolved in DMSO and then diluted with 10 mM PIPES. Thus, the treating solutions contained traces of DMSO ranging from 0.03 to 0.21% (v/v). These concentrations of DMSO did not affect light-induced chloroplast responses (results not shown). Control experiments were carried out using 10 mM PIPES and, additionally, 5 mM KNO_3 _(also in PIPES). The latter solution was used because Ca^2+ ^and Mg^2+ ^were applied as nitrates. No significant differences existed between chloroplast responses and/or cytoskeleton images in these two control solutions. Except for PIPES (Duchefa) all chemicals came from Sigma. The solutions were prepared with spectrochemically pure water, and their pH was adjusted to 6.8 with NaOH. They were stored in calcium-free plastic containers washed with concentrated HNO_3_, 10 mM EGTA and rinsed several times with spectrochemically pure water. Concentrations and incubation times were optimized in preliminary tests for each solution on the basis of clearly observable changes in the actin cytoskeleton and chloroplast movement. The chosen incubation periods ranged from 30 min to 3 h.

### Preparation of samples

The whole plant was adapted to darkness for at least 12 h before the experiment. The leaves were detached, the lower epidermis was removed and the tissue was cut into small pieces. After gentle infiltration with distilled water the pieces were stored in water for further usage. During storage, which never exceeded 12 h, no disturbances in the appearance of the AC or chloroplast responses were observed. Several samples at a time were infiltrated with a control or test solution and incubated as required, with constant slow mixing. All infiltrations were done in plastic syringes. Following incubation, some samples were placed on microscope slides to assess the AC organization, while other samples were used for photometric measurements. To prevent drying, the microscope preparations were enclosed in parafilm chambers sealed with silicon grease. All samples were prepared under green safe light and stored in the dark at room temperature.

### Confocal Microscopy

The fluorescence of GFP was visualized with the confocal microscope BioRad MRC 1024 (BioRad, Hercules, CA). Images were collected using a 60× (NA 1.4) PlanApo oil-immersion objective mounted on a Nikon microscope. Fluorescence was excited with blue light at 488 nm emitted by a 100 mW argon-ion air-cooled laser (ITL, USA). GFP fluorescence was viewed in the green channel, with the filter 540 DF30, and autofluorescence of chloroplasts – in the red channel, with the filter 585LP. The argon-ion laser was used at 10% (sporadically at 30%) of the maximum power for imaging.

The illumination was performed in the microscope or under a separate halogen lamp. For BL, the lamp was fitted with blue filter foil (λmax 424 nm, half-band width, 381–482 nm, Filmfabrik Wolfen, Wolfen, Germany). For RL (λmax 646 nm), the lamp was fitted with an RG1 filter, a C805 heat absorbing filter (Schott, Jena, Germany), and a dichroic short-pass filter (PZO, Warszawa, Poland). The applied fluence rates of blue and red light had equivalent quantum fluxes, weak blue (wB) 0.4 Wm^-2 ^and weak red (wR) 0.24 Wm^-2^, strong blue (SB) 10 Wm^-2 ^and strong red (SR) 6.7 Wm^-2^. The fluence rates were measured with a silicon photodiode calibrated against a LI-COR quantum meter (Li-Cor, Lincoln, NB, USA). Samples were irradiated for 60 min with weak and 20 min with strong light in the microscope. The effects of strong light were visible after 20 min; longer irradiation caused fading of fluorescence due to GFP photobleaching. The appearance of AC was checked before and immediately after every irradiation with the confocal microscope. The spongy mesophyll cells situated at least 3 cells away from the vessels were tested. The AC of cells situated near vessels and tracheids was less sensitive to the treatments applied in this study. Images were assembled from 3 to 15 optical sections collected with 0.5 μm steps. All the images presented show the AC at the periclinal walls (abaxial side) of mesophyll cells. No differences were observed in the structure of the AC at the adaxial side. Mitochondria were stained by 10 min incubation with TMRE (10 μM, tetramethyl rhodamine ethyl ester, Sigma) and the images were merged with those of GFP.

Each experimental variant was repeated in at least 3 independent series, with 3 to 6 images collected in each series.

### Image analysis and processing

To quantify changes in cytoskeleton architecture thickness of actin bundles in cytoplasm and order parameter of actin baskets at chloroplasts were calculated on single optical sections separately (i.e. 2D methods were used). Sections containing actin-GFP fluorescence and chloroplast autofluorescence were pre-processed using 3 × 3 hybrid median filter to suppress noise. Images corresponding to these two bands of fluorescence were segmented to isolate chloroplasts and AC using global thresholding. The threshold levels were calculated using Otsu algorithm [45] and all data from control experiments as an input.

In order to compute thickness of AFs, binary masks corresponding to chloroplasts were dilated (single pass, 3 × 3 uniform structuring element) and subtracted from the binary masks corresponding to total actin. The resulting images were convolved with gradient magnitude operator (gaussian derivative, σ = 1), thresholded and skeletonized in order to isolate edges. Average thickness of these AFs was calculated (on a section by section basis) by dividing the area (sum of respective cytoplasmic actin binary mask) by total length of edges (sum of respective skeletonized binary image). The thickness was typically greater in the top images of a stack (horizontal sections through actin layer) than in bottom images (vertical sections). Therefore, minimum and maximum thickness of AF section was estimated by taking, respectively, 1st and 99th percentile of data set corresponding to all optical sections (15–30 sections, 3–5 stacks) taken at the same experimental conditions. The maximum corresponded to thickness of AF bundles visible in the maximum z-projections of the confocal stacks (all images shown in this work). The minimum corresponded to the thickness measured in the plane orthogonal to the former. One should note that the minimum was in the range of 0.7 to 1.1 μm. under all conditions. These facts indicate consistent performance of AF bundle segmentation and measurement routine. Furthermore, one may postulate that the bundles were not circular and that the changes of their architecture were directional.

To quantify the sharpness (energy) of pattern of AF distribution in the baskets around chloroplasts the images corresponding to GFP were convolved with gradient magnitude operator (gaussian derivative, σ = 0.75). Average intensity in these images was calculated over areas marked by the binary masks corresponding to chloroplasts. The masks were calculated as described in the previous section, but merged chloroplasts were separated using watershed (performed on original images). The density was computed by taking percentiles (1, 5, 50, 95, 99) of the data set corresponding to the same experimental conditions.

### Photometric method

Chloroplast movements were measured with a custom-made double-beam photometer [46]. Monochromatic red light λ = 660 nm at a fluence-rate of 0,1 μmol m^-2^s^-1^(15 mWm^-2^), modulated with the frequency of 800 Hz served as the measuring light. Actinic light was obtained from a halogen lamp (100W, 12V). BL (λ_max _423 nm, half-band width, 86 nm) was obtained with a combination of filters, BG12, BG23, GG13 and a heat-absorbing C805 (all from Schott, Jena, Germany). Light-induced chloroplast responses were recorded as changes in light transmission through the leaf pieces.

Only samples with similar levels of initial transmission after dark adaptation (between 38 and 41%) were selected. The sample was placed on a glass slide in a drop of the incubation medium. It was closed in a miniature chamber formed by a saran-wrap cover stretched over a metal ring mounted on a parafilm seal. The dark-adapted leaf sample was illuminated perpendicular to its adaxial surface with weak blue light (0.4 Wm^-2^) followed by strong blue light (10 Wm^-2^) for 45 minutes with each fluence rate. The following parameters were measured/calculated to characterize the chloroplast responses, 1) amplitudes – transmission changes after 45 min of each response 2) velocities – first derivatives of the initial (lasting about 10 min) linear fragments of the respective transmission curves (see Fig. 1A).

## Abbreviations

**ABPs**: actin binding proteins; **AC**: actin cytoskeleton; **AFs**: actin filaments; **GFP**: green fluorescent protein; Δ**T**: transmission change; **TFP**: trifluoperazine; **TMRE**: tetramethyl rodamine ethyl ester; **wL**: weak light; **wBL/wRL**: weak blue/red light; **SL**: strong light; **SBL/SRL**: strong blue/red light: **WM**: wortmannin.

## Authors' contributions

AAM created the transgenic plant, designed the study, carried out the experiments and prepared the manuscript. TB carried out the quantitative image analysis. HG conceived of the study, supervised the collection, analysis and interpretation of data, and helped in preparing the manuscript. All authors read and approved the final manuscript.

## Supplementary Material

Additional file 1 **Energy of actin distribution pattern in F-actin baskets surrounding chloroplasts**. Sharpness (energy) of actin distribution pattern in the baskets at the chloroplasts in control (Ctrl), and in the presence of 5 mM Ca^+2 ^or Mg^+2^, in cells treated for 2 h with the ions only, or pre-treated with 1 mM EGTA or 20 μM TFP for 30 min. The network energy was measured in cells adapted to darkness (Dad, gray bars) and in cells illuminated with strong (SB) or weak (wB) blue light (bright and dark blue bars, respectively) and with strong (SB) or weak (wR) red light (bright and dark red bars, respectively). Higher energy corresponds to higher inhomogeneity of actin structure. The energy was computed in arbitrary units (A.U., see Materials and Methods) as 1st, 50th (median) and 99th percentile of the data corresponding to a population of chloroplasts in each set of experimental conditions. Error bars represent 95% confidence intervals. One may note that energy corresponding to lowest (1st) percentile was non-zero and similar in all the conditions. This fact indicates the presence of constitutive non-uniform actin distribution (structure) at the chloroplasts. The disruption of actin baskets under strong irradiation (red and blue) was manifested clearly only at the highest (99th) percentile of the pattern energy data. Therefore, it may be postulated that filaments (and their edges), which contribute to this effect, occupy only minor fraction of chloroplast surface. Click here for file

Additional file 2 **Reorganization of F-actin during strong blue light irradiation**. Changes in reorganization of actin bundles and in chloroplast distribution in lower mesophyll cells during SBL irradiation. The first image shows the weak white light-adapted tissue. Images B and C were collected after 10 and 20 min respectively. The avoidance response of chloroplasts needs approximately 1.5 h to be completed. Therefore, only partial redistribution of chloroplasts towards profile position is noticeable in several cells (marked with arrows). Scale bar, 10 μm. Click here for file

Additional file 3 **Reorganization of wide F-actin strands in a tobacco cell adjacent to the vascular bundle**. Dynamic reorganization of wide F-actin strands in a tobacco cell adjacent to the vascular bundle irradiated with SBL. Time series collected every 2 min. Click here for file

Additional file 4 **Reorganization of wide F-actin strands in tobacco mesophyll cells**. Time series collected every 2 min. Click here for file

Additional file 5 **Reorganization of wide F-actin strands in tobacco mesophyll cells**. Note the dynamic movement of nucleus in the upper cell. Time series collected every 2 min. Click here for file

Additional file 6 **Reversibility of the "diffusion" effect induced by strong blue light**. Single confocal scans of a cortical part of the same mesophyll cell after consecutive irradiations with continuous SBL (20 min) and wBL (60 min). (A) Branched actin network in the dark-adapted cell; (B) Diffuse F-actin forming single widened strands after SBL irradiation (marked with arrow); (C) Reconstruction of distinct bundles (marked with arrows) by wBL. Scale bars, 10 μm. Click here for file

Additional file 7 **The effect of strong light on F-actin in cells with modified Ca^2+^/Mg^2+ ^levels**. Diffuse widened strands appearing after exposure to continuous strong blue (left panels) and red light (right panels). Prior to irradiation, the samples were incubated with (A) 5 mM Ca^2+ ^for 2 h, (B) 5 mM Mg^2+ ^for 2 h, (C, D) 1 mM EGTA for 45 min, (E, F) 20 μM solution of calmodulin inhibitor TFP for 45 min, (G) 20 μM TFP for 45 min followed by 5 mM Ca^2+ ^for 2 h, (H) 20 μM TFP for 45 min followed by 5 mM Mg^2+ ^for 2 h. Scale bars, 10 μm. Click here for file

Additional file 8 **Combined action of EGTA and calcium ionophore on the cytoskeleton**. Effect of 1 mM EGTA + calcium ionophore A23187 on the actin cytoskeleton, after 15 (A), 30 (B) and 45 min (C). Note the transient formation of baskets around the chloroplasts. The filaments making baskets developed slowly over the first 30 min of the incubation period (b) and gradually faded away over the next 30 min. Scale bars, 10 μm. Click here for file

## References

[B1] Grolig F, Hussey PJ (2004). Organelle movements: transport and positioning. The plant cytoskeleton in cell differentiation and development.

[B2] Drøbak BK, Franklin-Tong VE, Staiger CJ (2004). The role of the actin cytoskeleton in plant cell signaling. New Phytologist.

[B3] Haupt W, Scheuerlein R (1990). Chloroplast movement. Plant Cell Environ.

[B4] Sakamoto K, Briggs WR (2002). Cellular and subcellular localization of phototropin 1. Plant Cell.

[B5] Harada A, Sakai T, Okada K (2003). Phot1 and phot2 mediate blue light-induced transient increase in cytosolic Ca^2+ ^differently in *Arabidopsis *leaves. Proc Natl Acad Sci USA.

[B6] Jarillo JA, Gabryś H, Capel J, Alonso JM, Ecker JR, Cashmore AR (2001). Phototropin-related NPL-1 controls chloroplast relocation induced by blue light. Nature.

[B7] Sakai T, Kagawa T, Kasahara M, Swartz TE, Christie JM, Briggs WR, Wada M, Okada K (2001). *Arabidopsis *nph1 and npl1, Blue light receptors that mediate both phototropism and chloroplast relocation. Proc Natl Acad Sci USA.

[B8] Sato Y, Wada M, Kadota A (2001). Choice of tracks, microtubules and/or actin filaments for chloroplast photo-movement is differentially controlled by phytochrome and a blue light receptor. J Cell Sci.

[B9] Takagi S (2003). Actin-based photo-orientation movement of chloroplasts in plant cells. J Exp Biol.

[B10] Malec P, Rinaldi RA, Gabryś H (1996). Light-induced chloroplast movements in *Lemna trisulca*. Identification of the motile system. Plant Sci.

[B11] Wojtaszek P, Anielska-Mazur A, Gabryś H, Baluška F, Volkmann D (2005). Recruitment of myosin VIII towards plastid surfaces is root-cap specific and provides the evidence for actomyosin involvement in root osmosensing. Funct Plant Biol.

[B12] Krzeszowiec W, Gabryś H (2007). Blue light-induced reorganization of myosins in *Arabidopsis *thaliana. Plant Signal Behav.

[B13] Kandasamy MRB, Meagher RB (1999). Actin-organelle interaction, association with chloroplast in *Arabidopsis *leaf mesophyll cells. Cell Motil Cytoskeleton.

[B14] Gabryś H (2004). Blue light-induced orientation movements of chloroplasts in higher plants. Recent progress in the study of their mechanisms. Acta Physiol Plant.

[B15] Spalding EP, Folta KM (2005). Illuminating topics in plant photobiology. Plant Cell Environ.

[B16] Sanders D, Brownlee C, Harper JF (1999). Communicating with calcium. Plant Cell.

[B17] Sanders D, Pelloux J, Brownlee C, Harper JF (2002). Calcium at the crossroads of signaling. Plant Cell.

[B18] Snedden WA, Fromm H (2001). Calmodulin as a versatile calcium signal transducer in plants. New Phytol.

[B19] White PJ, Broadley MR (2003). Calcium in plants. Ann Bot (Lond).

[B20] Baum G, Long JC, Jenkins GI, Trewavas AJ (1999). Stimulation of the blue light phototropic receptor NPH1 causes a transient increase in cytosolic Ca^2+^. Proc Natl Acad Sci USA.

[B21] Babourina O, Newman I, Shabala S (2002). Blue light-induced kinetics of H^+ ^and Ca^2+ ^fluxes in etiolated wild-type and phototropin-mutant *Arabidopsis *seedlings. Proc Natl Acad Sci USA.

[B22] Stoelzle S, Kagawa T, Wada M, Hedrich R, Dietrich P (2003). Blue light activates calcium-permeable channels in *Arabidopsis *mesophyll cells via phototropin signaling pathway. Proc Natl Acad Sci USA.

[B23] Takagi S (1997). Photoregulation of cytoplasmic streaming, Cell biological dissection of signal transduction pathway. J Plant Res.

[B24] Tlałka M, Gabryś H (1993). Influence of calcium on blue-light-induced chloroplast movement in *Lemna trisulca *L. Planta.

[B25] Tlałka M, Fricker M (1999). The role of calcium in blue-light-dependent chloroplast movement in *Lemna trisulca *L. Plant J.

[B26] Grabalska M, Malec P (2004). Blue light-induced chloroplast reorientations in Lemna trisulca L. (duckweed) are controlled by two separable cellular mechanisms as suggested by diffrent sensitivity to wortmannin. Photochem Photobiol.

[B27] Krzeszowiec W, Rajwa B, Dobrucki J, Gabryś H (2007). Actin cytoskeleton in *Arabidopsis thaliana *under blue and red light. Biol Cell.

[B28] Timmers ACJ, Niebel A, Balague C, Dagkesamanskaya A (2002). Differential localisation of GFP fusions to cytoskeleton-binding proteins in animal, plant, and yeast cells. Protoplasma.

[B29] Zurzycki J (1960). Studies on the centrifugation of chloroplasts in *Lemna trisulca*. Acta Soc Bot Pol.

[B30] Kadota A, Wada M (1992). Photoinduction of formation of circular structures by microfilaments on chloroplasts during intracellular orientation in protonemal cells of the fern *Adiantum-capillus-veneris*. Protoplasma.

[B31] Augustynowicz J, Lekka M, Burda K, Gabryś H (2001). Correlation between chloroplast motility and elastic properties of tobacco mesophyll protoplasts. Acta Physiol Plant.

[B32] Kengen HMP, de Graaf BHJ (1991). Microtubules and actin filaments co-localize extensively in non-fixed cells of tobacco. Protoplasma.

[B33] Snowman BN, Kovar DR, Shevchenko G, Franklin-Tong VE, Staiger CJ (2002). Signal-mediated depolymerization of actin in pollen during the self-incompatibility response. Plant Cell.

[B34] Frieden C (1983). Polymerization of actin, Mechanism of the Mg^2+^-induced process at pH 8 and 20°C. Proc Natl Acad Sci USA.

[B35] Carlier MF, Pantaloni D, Korn ED (1986). The effects of Mg^2+ ^at the high-affinity and low affinity sites on the polymerization of actin and associated ATP hydrolysis. J Biol Chem.

[B36] Carlier MF (1990). Actin polymerization and ATP hydrolysis. Adv Bioph.

[B37] Estes JE, Selden LA, Kinosian HJ, Gershman LC (1992). Tightly-bound divalent cation of actin. J Muscle Res Cell Motil.

[B38] Strzelecka-Gołaszewska H, Woźniak A, Hult T, Lindberg U (1996). Effects of the type of divalent cation, Ca^2+ ^or Mg^2+^, bound at the high affinity site and of the ionic composition of the solution on the structure of F-actin. Biochem J.

[B39] Kadota A, Wada M (1992). Photoorientation of chloroplasts in protonemal cells of the fern *Adiantum *as analized by use of a video-tracking system. J Plant Res.

[B40] Shabala S, Hariadi Y (2005). Effect of magnesium availability on the activity of plasma membrane ion transporters and light-induced responses from broad bean leaf mesophyll. Planta.

[B41] Murphy E (2000). Mysteries of magnesium homeostasis. Circ Res.

[B42] Shaul O (2002). Magnesium transport and function in plants, the tip of the iceberg. BioMetals.

[B43] Gardner RC (2003). Genes for magnesium transport. Curr Opin Plant Biol.

[B44] Jung JY, Kim YW, Kwak JM, Hwang JU, Young Y, Schroeder JI, Hwang I, Lee Y (2002). Phosphatidylinositol 3- and 4-phosphate are required for normal stomatal movements. Plant Cell.

[B45] Otsu N (1979). A threshold selection method from gray-scale histogram. IEEE Trans Systems, Man, and Cybernetics.

[B46] Walczak T, Gabryś H (1980). New type of photometer for measurements of transmission changes corresponding to chloroplast movements in leaves. Photosynthetica.

