# Supplementary figures and images for: In vivo reorganization of the actin cytoskeleton in leaves of Nicotiana tabacum L. transformed with plastin-GFP. Correlation with light-activated chloroplast responses
Source: BMC Plant Biol. 2009 May 29;9:64. doi: 10.1186/1471-2229-9-64 (PMC2702303; doi:10.1186/1471-2229-9-64)

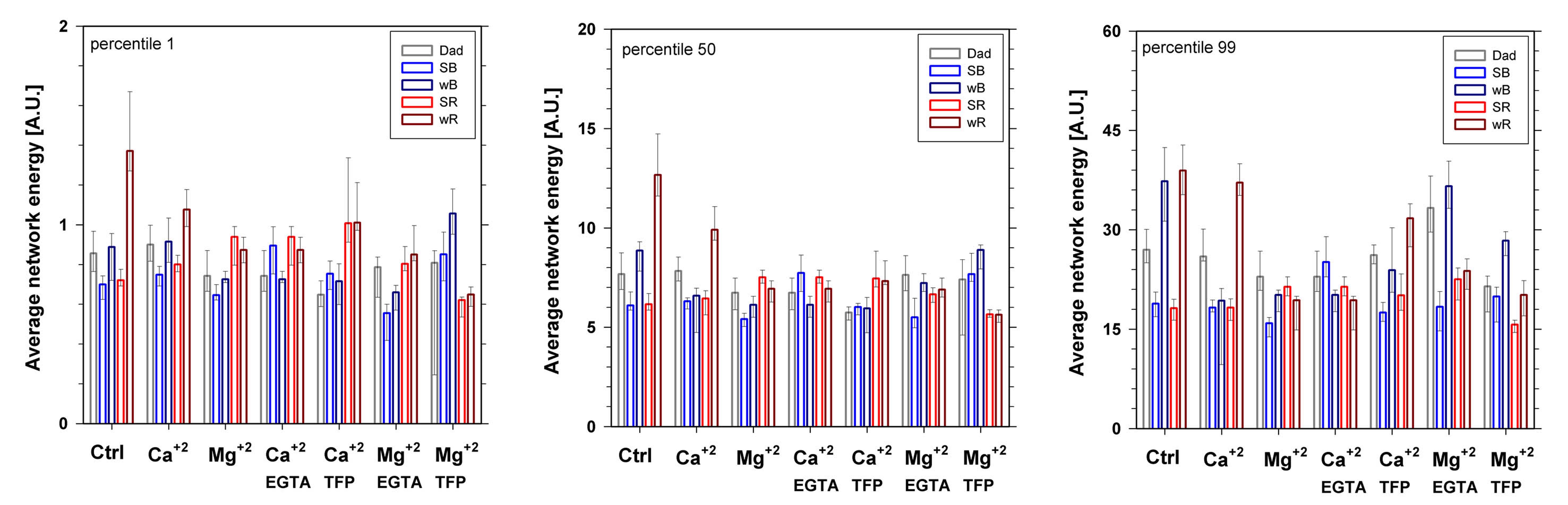

Supplement: Additional file 1 — Energy of actin distribution pattern in F-actin baskets surrounding chloroplasts. Sharpness (energy) of actin distribution pattern in the baskets at the chloroplasts in control (Ctrl), and in the presence of 5 mM Ca+2 or Mg+2, in cells treated for 2 h with the ions only, or pre-treated with 1 mM EGTA or 20 μM TFP for 30 min. The network energy was measured in cells adapted to darkness (Dad, gray bars) and in cells illuminated with strong (SB) or weak (wB) blue light (bright and dark blue bars, respectively) and with strong (SB) or weak (wR) red light (bright and dark red bars, respectively). Higher energy corresponds to higher inhomogeneity of actin structure. The energy was computed in arbitrary units (A.U., see Materials and Methods) as 1st, 50th (median) and 99th percentile of the data corresponding to a population of chloroplasts in each set of experimental conditions. Error bars represent 95% confidence intervals. One may note that energy corresponding to lowest (1st) percentile was non-zero and similar in all the conditions. This fact indicates the presence of constitutive non-uniform actin distribution (structure) at the chloroplasts. The disruption of actin baskets under strong irradiation (red and blue) was manifested clearly only at the highest (99th) percentile of the pattern energy data. Therefore, it may be postulated that filaments (and their edges), which contribute to this effect, occupy only minor fraction of chloroplast surface. [file 1471-2229-9-64-S1.jpeg]

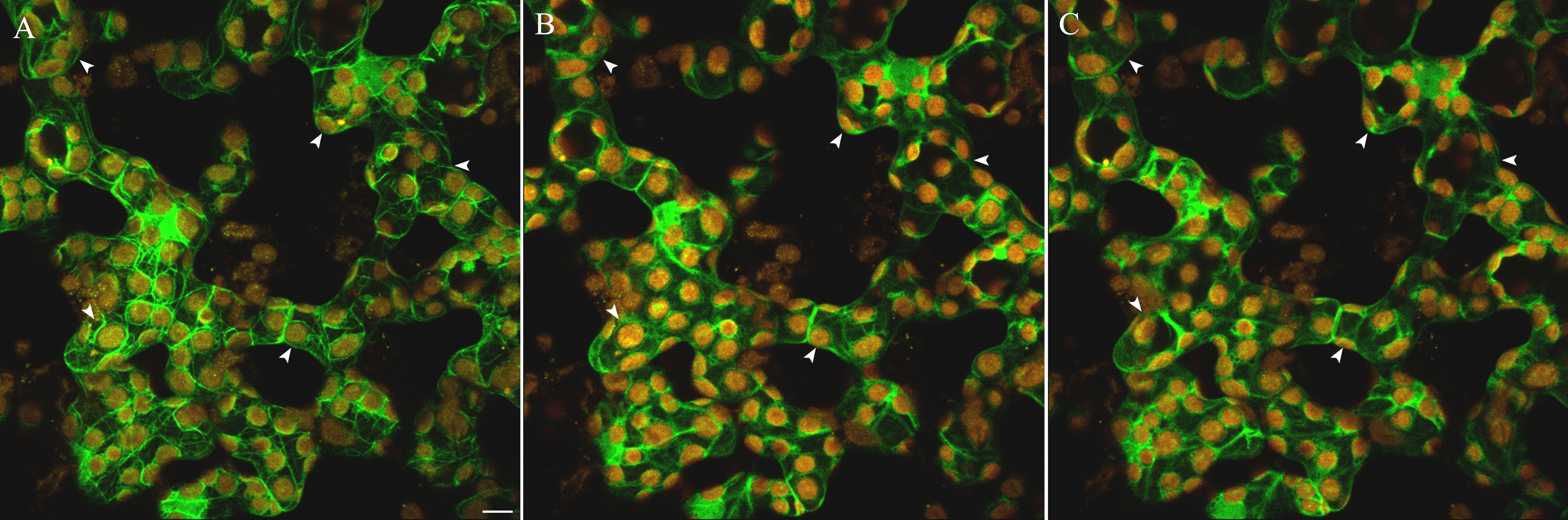

Supplement: Additional file 2 — Reorganization of F-actin during strong blue light irradiation. Changes in reorganization of actin bundles and in chloroplast distribution in lower mesophyll cells during SBL irradiation. The first image shows the weak white light-adapted tissue. Images B and C were collected after 10 and 20 min respectively. The avoidance response of chloroplasts needs approximately 1.5 h to be completed. Therefore, only partial redistribution of chloroplasts towards profile position is noticeable in several cells (marked with arrows). Scale bar, 10 μm. [file 1471-2229-9-64-S2.jpeg]

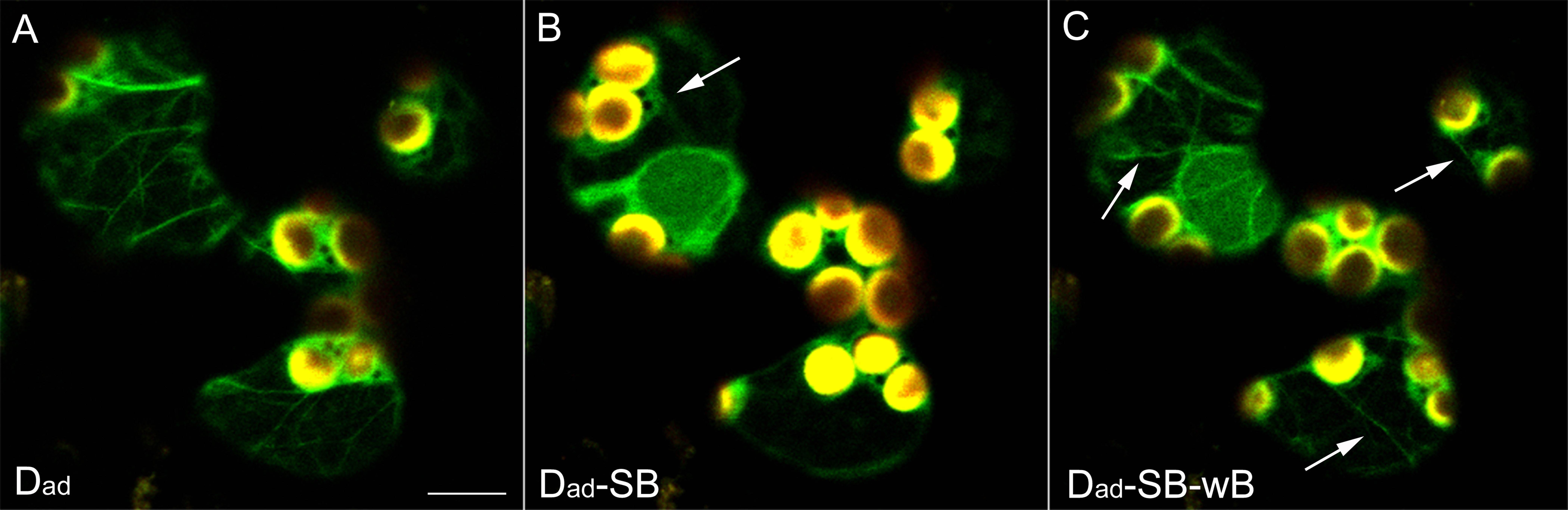

Supplement: Additional file 6 — Reversibility of the "diffusion" effect induced by strong blue light. Single confocal scans of a cortical part of the same mesophyll cell after consecutive irradiations with continuous SBL (20 min) and wBL (60 min). (A) Branched actin network in the dark-adapted cell; (B) Diffuse F-actin forming single widened strands after SBL irradiation (marked with arrow); (C) Reconstruction of distinct bundles (marked with arrows) by wBL. Scale bars, 10 μm. [file 1471-2229-9-64-S6.jpeg]

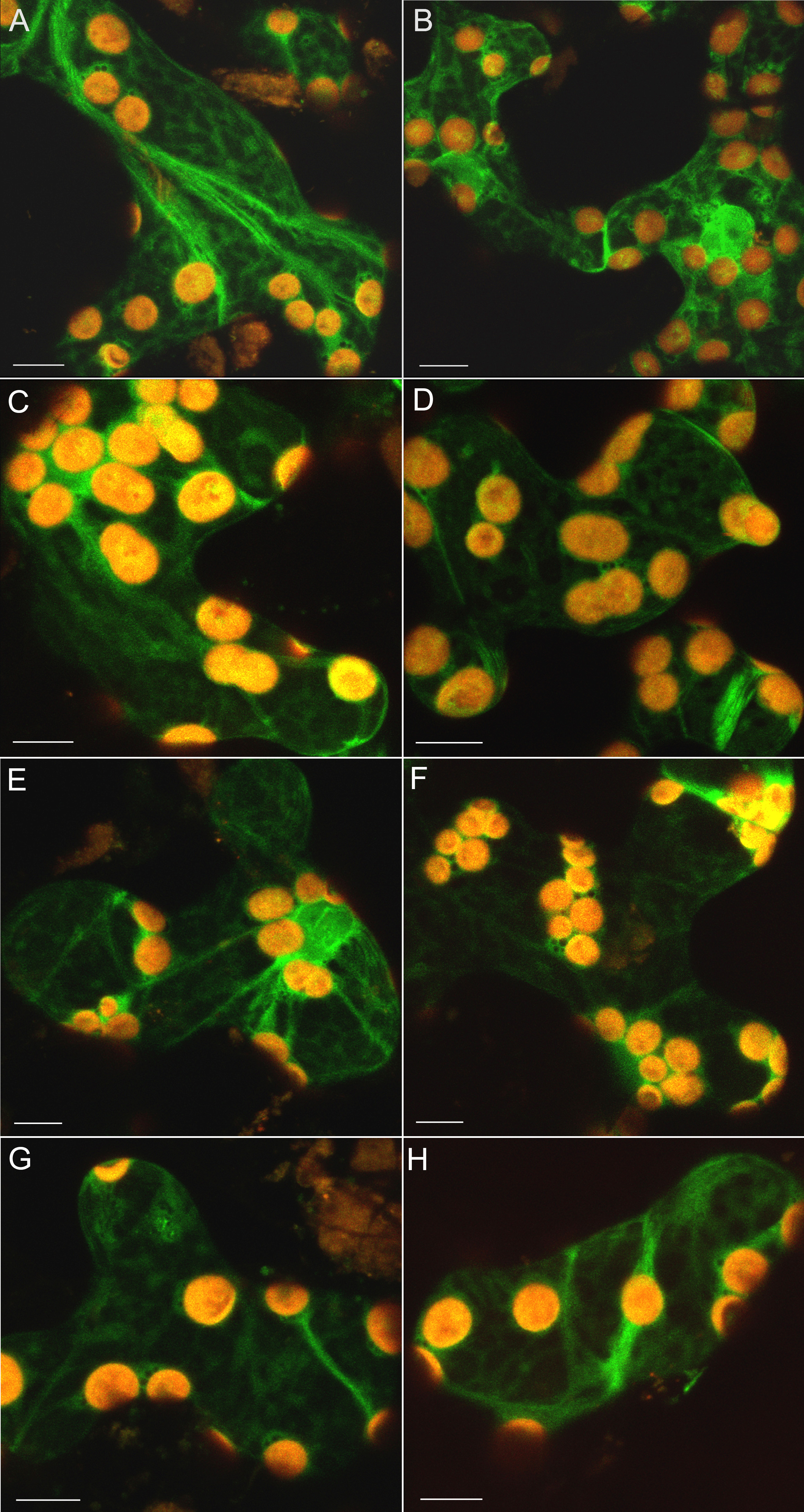

Supplement: Additional file 7 — The effect of strong light on F-actin in cells with modified Ca2+/Mg2+ levels. Diffuse widened strands appearing after exposure to continuous strong blue (left panels) and red light (right panels). Prior to irradiation, the samples were incubated with (A) 5 mM Ca2+ for 2 h, (B) 5 mM Mg2+ for 2 h, (C, D) 1 mM EGTA for 45 min, (E, F) 20 μM solution of calmodulin inhibitor TFP for 45 min, (G) 20 μM TFP for 45 min followed by 5 mM Ca2+ for 2 h, (H) 20 μM TFP for 45 min followed by 5 mM Mg2+ for 2 h. Scale bars, 10 μm. [file 1471-2229-9-64-S7.jpeg]

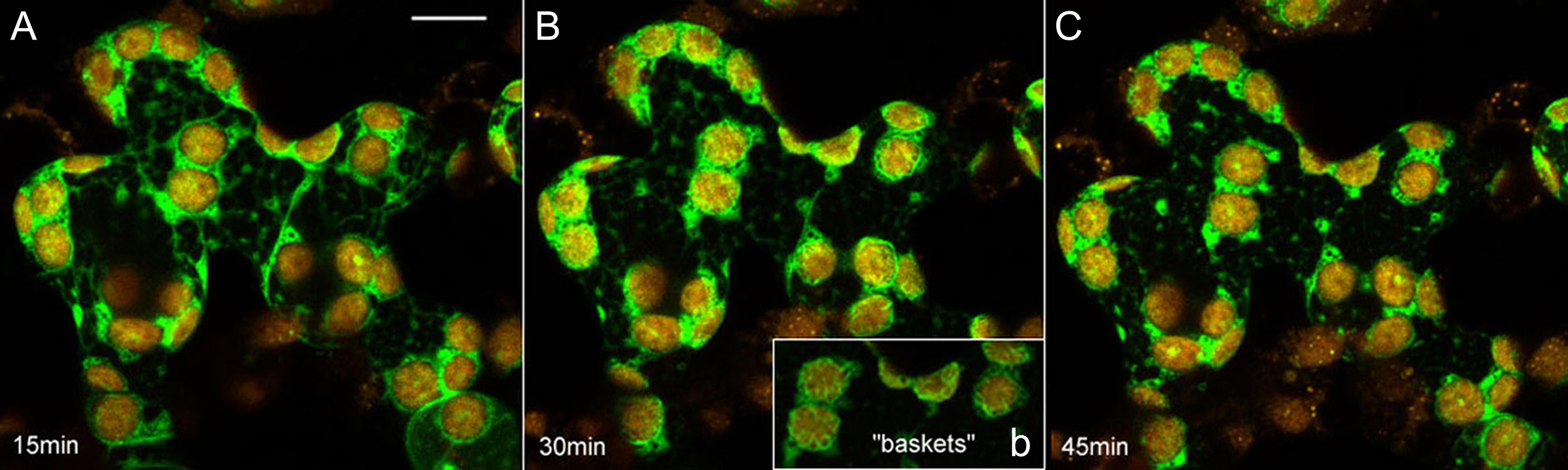

Supplement: Additional file 8 — Combined action of EGTA and calcium ionophore on the cytoskeleton. Effect of 1 mM EGTA + calcium ionophore A23187 on the actin cytoskeleton, after 15 (A), 30 (B) and 45 min (C). Note the transient formation of baskets around the chloroplasts. The filaments making baskets developed slowly over the first 30 min of the incubation period (b) and gradually faded away over the next 30 min. Scale bars, 10 μm. [file 1471-2229-9-64-S8.jpeg]
